# Supplementary material for: Mechanistic insights into the role of prenyl-binding protein PrBP/δ in membrane dissociation of phosphodiesterase 6
Source: Nat Commun. 2018 Jan 8;9:90. doi: 10.1038/s41467-017-02569-y (PMC5758567; doi:10.1038/s41467-017-02569-y)
Supplement: Supplementary file 1 — Supplementary Information [file 41467_2017_2569_MOESM1_ESM.pdf]

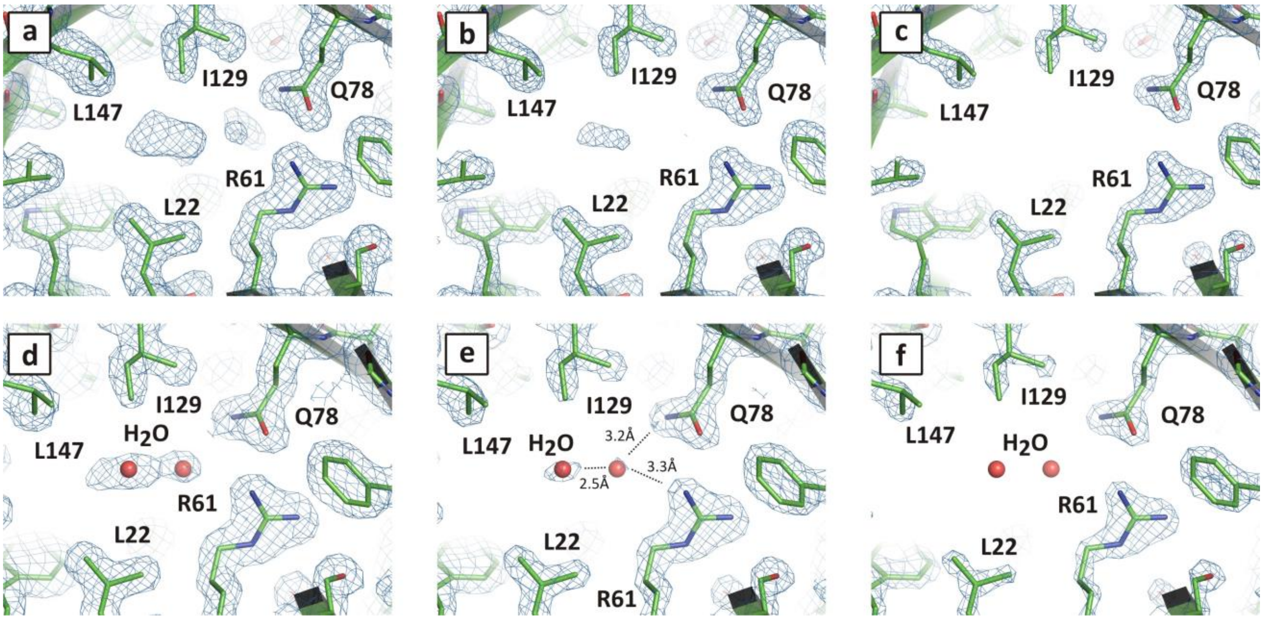

Supplementary Figure 1 │2m*Fo*-D*Fc* omit map of the apo-PrBP/δ binding cavity

Apo-PrBP/δ is shown as cartoon-stick representation (green color). In figure **(a-c)** electron densities were shown as simple 2m*Fo*-D*Fc* omit map calculated with the program PHENIX[1] and contoured at **(a)** 1.0 σ, **(b)** 1.5 σ and **(c)** 2.0 σ. In figures **(d-f)** electron densities were shown as 2m*Fo*-D*Fc* simulated annealing omit map calculated with the program PHENIX[1] for omitted water molecules and contoured at **(d)** 1.0 σ, **(e)** 1.5 σ and **(f)** 2.0 σ.

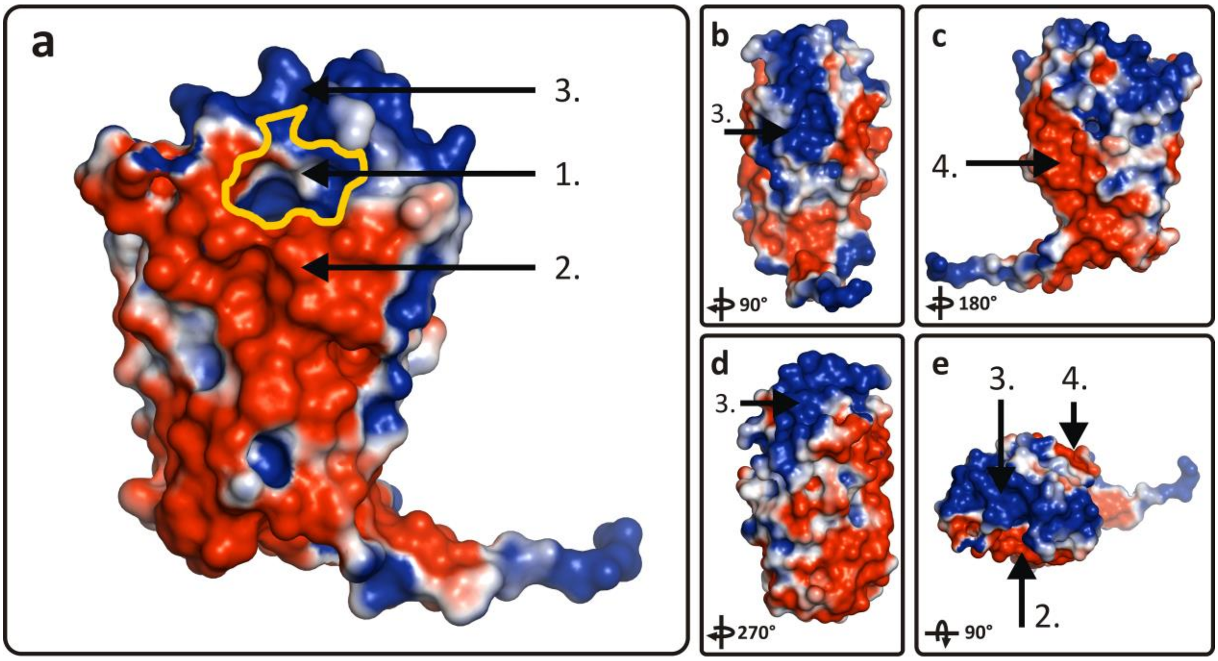

Supplementary Figure 2 | Electrostatic surface representation of apo-PrBP/δ: Electrostatic surface potentials were calculated using the program APBS with the non-linear Poisson-Boltzmann equation and contoured at +/- 2kT e^{-1}.[2] Negatively and positively charged surface areas are colored in red and blue, respectively. To elucidate the entrance of the cavity a yellow edge was introduced manually. In **(a)** electrostatic surface representation of apo-PrBP/δ is shown in front side perspective with (1) entrance of the cavity, (2) large negative-charged surface at the side and (3) positive-charged surface potential at the top. **(b)** Apo-PrBP/δ is rotated by 90° on the y-axis; **(c)** back-side of (b), **(d)** apo-PrBP/δ is rotated by 270° on the y-axis and **(e)** apo-PrBP/δ rotated by 90° on the x-axis.

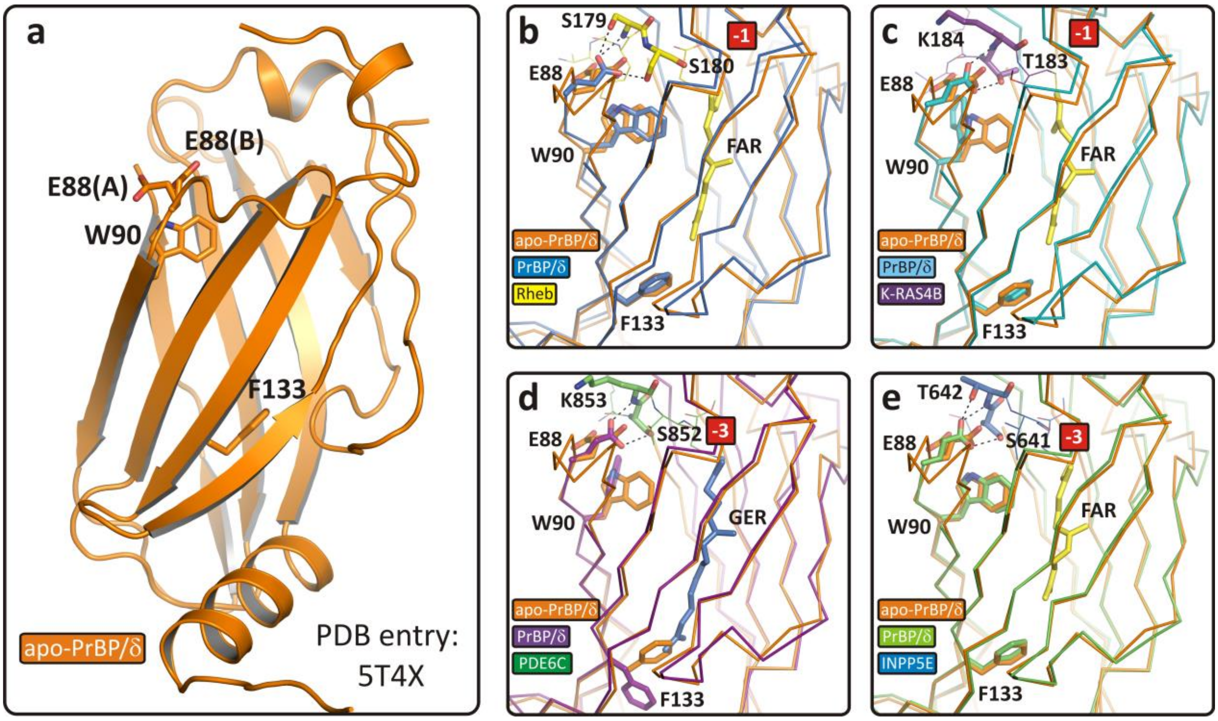

Supplementary Figure 3 │ Superposition of crystal structures of apo-PrBP/δ and cargo-bound PrBP/δ's. **(a)** Apo-PrBP/δ is shown as cartoon representation (orange). Glu88 (in double conformation E88 (A/B)), Trp90 and Phe133 are highlighted as sticks. **(b)** Superposition of apo-PrBP/δ (orange) and the complex of PrBP/δ (blue) bound to farnesylated Rheb (yellow) (PDB entry 3T5G). **(c)** Superposition of apo-PrBP/δ (orange) and the complex of PrBP/δ (light-blue) bound to farnesylated K-RAS4B (purple) (PDB entry 3TAR). **(d)** Superposition of apo-PrBP/δ (orange) and the complex of PrBP/δ (purple) bound to geranylgeranylated PDE6C-peptide (dark green) (PDB entry 5E8F). **(e)** Superposition of apo-PrBP/δ (orange) and the complex of PrBP/δ (green) bound to farnesylated INPP5E-peptide (blue) (PDB entry 5F2U). The farnesyl- and the geranylgeranyl moiety are depicted as yellow and blue sticks, respectively. **(b-e)** PrBP/δ structures are shown in ribbon representation and key residues (Glu88, Trp90 and Phe133) for cargo-binding and cavity adaptation are represented as sticks. Amino acids of the cargo involved in hydrogen bonding with Glu88 are also depicted as sticks. The release of the cargo is regulated by the displacement factor (DF). Arl2 and Arl3 are known

to act as DF on PrBP/δ, depending on different cargos to be bound. Fansa and coworkers associate the displacement factors specificity with the different binding mode of the cargo proteins. While Arl2 is identified as DF for low affinity binder (as in **(b)** and **(c))**, where Glu88 binds *e.g.* to cargo residue -1 numbered from last cysteine, Arl3 act on high affinity complexes shown in **(d)** and **(e)** that reveal hydrogen bonding between Glu88 (PrBP/δ) with *e.g.* -3 residue from last cysteine of the cargo protein.

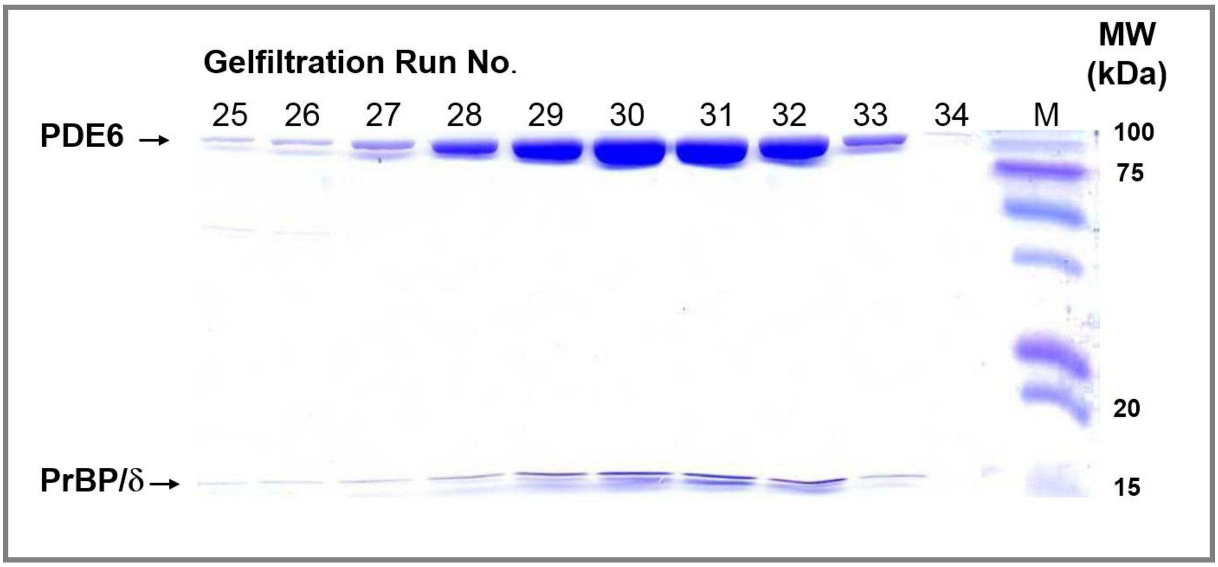

Supplementary Figure 4│ SDS-polyacrylamide gel electrophoresis of size-exclusion chromatography runs for PDE6-PrBP/δ$_2$.

Fractions of the size-exclusion chromatography run (PDE6-PrBP/δ$_2$) are showing protein bands for PDE6 catalytic subunits (~ 90 kDa) and PrBP/δ (~17 kDa).

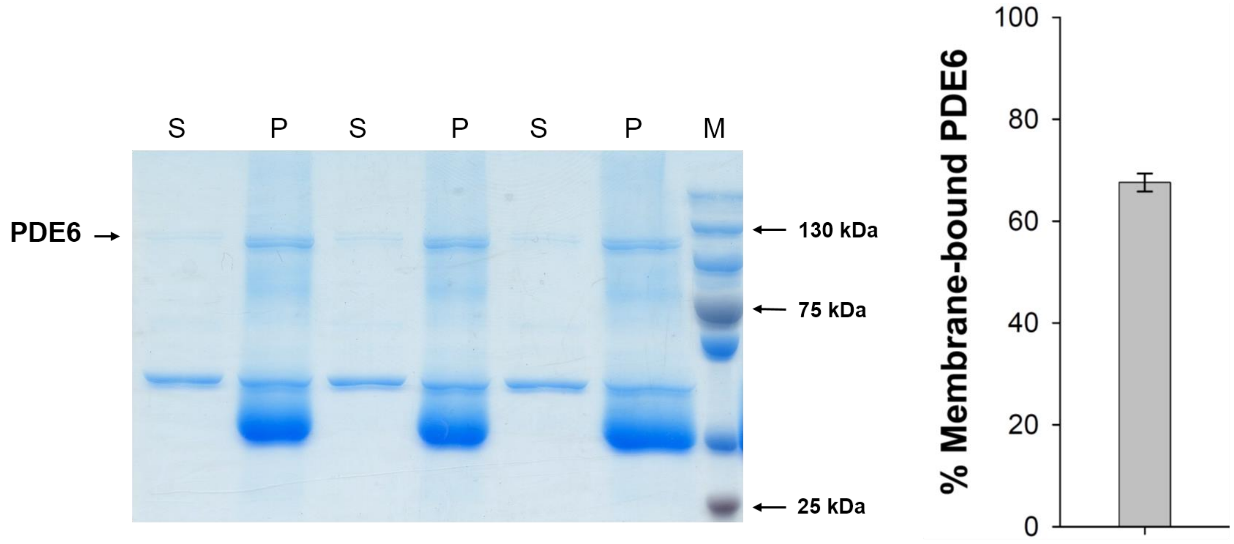

Supplementary Figure 5│ SDS-polyacrylamide gel electrophoresis of pull-down assay shows membrane-bound PrBP/δ. Samples containing PDE6 were parted into soluble (S) membrane-bound (P) PDE6 fractions by subjecting them to centrifugation.

Supplementary References

1. Adams PD, *et al.* PHENIX: a comprehensive Python-based system for macromolecular structure solution. *Acta crystallographica Section D, Biological crystallography* **66**, 213-221 (2010).

2. Baker NA, Sept D, Joseph S, Holst MJ, McCammon JA. Electrostatics of nanosystems: application to microtubules and the ribosome. *Proceedings of the National Academy of Sciences of the United States of America* **98**, 10037-10041 (2001).
